# *Priestia megaterium* Metabolism: Isolation, Identification of Naringenin Analogues and Genes Elevated Associated with Nanoparticle Intervention

**DOI:** 10.3390/cimb45080424

**Published:** 2023-08-14

**Authors:** Nada S. Al-Theyab, Hatem A. Abuelizz, Gadah A. Al-Hamoud, Ahmad Aldossary, Mingtao Liang

**Affiliations:** 1 School of Biomedical Science and Pharmacy, University of Newcastle, Callaghan, NSW 2308, Australia; nada.altheyab@uon.edu.au; 2 Department of Pharmaceutical Chemistry, College of Pharmacy, King Saud University, Riyadh 11451, Saudi Arabia; 3 Department of Pharmacognosy, College of Pharmacy, King Saud University, Riyadh 11451, Saudi Arabia; galhamoud@ksu.edu.sa; 4 Wellness and Preventative Medicine Institute, Health Sector, King Abdulaziz City for Science and Technology (KACST), Riyadh 11442, Saudi Arabia; aaldossary@kacst.edu.sa

**Keywords:** gold nanoparticles, *Priestia megaterium*, naringenin analogues, biosynthetic pathways, transcriptome analysis, drug discovery

## Abstract

The impact of gold nanoparticles (AuNPs) on the biosynthetic manipulation of *Priestia megaterium* metabolism where an existing gene cluster is enhanced to produce and enrich bioactive secondary metabolites has been studied previously. In this research, we aimed to isolate and elucidate the structure of metabolites of compounds 1 and 2 which have been analyzed previously in *P. megaterium* crude extract. This was achieved through a PREP-ODS C18 column with an HPLC-UV/visible detector. Then, the compounds were subjected to nuclear magnetic resonance (NMR), electrospray ionization mass spectrometry (ESI-MS), and Fourier-transform infrared spectroscopy (FT-IR) techniques. Furthermore, bioinformatics and transcriptome analysis were used to examine the gene expression for which the secondary metabolites produced in the presence of AuNPs showed significant enhancement in transcriptomic responses. The metabolites of compounds 1 and 2 were identified as daidzein and genistein, respectively. The real-time polymerase chain reaction (RT-PCR) technique was used to assess the expression of three genes (*csoR*, *CHS*, and *yjiB*) from a panel of selected genes known to be involved in the biosynthesis of the identified secondary metabolites. The expression levels of two genes (*csoR* and *yijB*) increased in response to AuNP intervention, whereas *CHS* was unaffected.

## 1. Introduction

Trace metals can function as effectors of metalloregulators or as cofactors of natural product biosynthetic enzymes in *Streptomyces* [1]. Additionally, they can influence the growth and morphology of *Streptomyces*. Recently, many metal ions have been studied for their ability to regulate secondary metabolite production in various microbes [2]. These metal ions can be involved in the initiation, activation, control, and inhibition of a variety of microbiological pathways as well as the interactions between soil, plants, and microbes. They are necessary for several metabolic processes, such as the electron transport chain, photosynthesis, and the transport and storage of various metabolites [3]. For example, copper ions can stimulate differentiation and antibiotic synthesis in *Streptomyces coelicolor* [4]. They can serve as a crucial cofactor in many enzymes and electron transport proteins through their redox activity [5]. As there is a low intracellular demand for Cu^+^, some microbes express efflux systems or excrete at least one kind of metal-binding metallothionein to control the internal levels of this ion. These metalloregulators, such as CopY, CsoR, and YcnK, are called copper-sensing transcriptional regulators and are mainly present in Gram-positive bacteria [6,7]. Most bacteria also produce P-type copper export ATPases (CopA) as defense mechanisms when copper concentrations are high [7]. It has been found that CsoR from *Thermus thermophilus* binds to several different metal ions in vitro, including Cu^+^, Cu^2+^, Zn^2+^, Cd^2+^, Ag^+^, and Ni^2+^, and allows the release of CsoR from the copper chaperone (*copZ*) promoter. Both copper and zinc ions can strongly upregulate *copZ*–*csoR*–*copA* expression in vivo [7,8]. Additionally, genomic modulation had been observed in *Saccharomyces cerevisiae* when exposed to silver ions and nanoparticles (AgNPs). The transcriptome analysis demonstrated that, in comparison to silver ions, AgNPs improved copper transport and other genes, and the majority of these AgNPs affected genes playing important roles in the metabolism [9]. There have been only a few reports of NPs’ effect on microorganisms’ growth characteristics and secondary metabolite profiles. Liu et al. studied the antibiotic production of *S. coelicolor M145* in the presence of copper oxide (CuO) NPs. They found that a low concentration of CuONPs increased the production of actinorhodin (ACT) antibiotics, whereas high concentrations inhibited this process. It was also observed that increased production of antibiotics was correlated with smaller sizes of CuO particles. Further, the transcriptional analysis demonstrated that the ACT biosynthetic gene cluster was significantly upregulated after exposure to CuONPs [10]. In another study, aluminum oxide nanoparticles (Al_2_O_3_NPs) were also shown to have a similar elicitor activity for secondary metabolism, while bulk particles had a low impact on the antibiotic production [11]. In recent years, increasing studies have demonstrated the role of NPs as an elicitor for inducing the expression of genes involved in the biosynthesis of secondary metabolites. Although many efforts are still required to elucidate the mechanism, an effect of NPs on cytochrome P450 enzyme overexpression involved in secondary metabolite biosynthesis has been proposed. The effect of cytochrome P450 (CYP) enzymes on metabolite biosynthesis has been widely explored in plants. For example, CYP71B6 in Arabidopsis was found to have a biological impact on the biosynthesis of defense-related indolic compounds upon treatment with silver nitrate (AgNO_3_) compared to untreated leaves, indicating that CYP71B6 is involved in the biosynthesis of ICOOH derivatives [12]. In another study, lipopolysaccharides (LPSs) have been investigated as a trigger for activating signal transduction pathways involved in the defense metabolites of *Arabidopsis thaliana*. The findings showed that the LPS perception triggered the enhanced expression and activity of CYP79B2/B3, leading to variation in the biosynthesis of specialized secondary metabolites [13]. The CYP450 enzymes are critical enzymes in the natural product biosynthetic pathway; their gene sequences have recently been discovered in bacteria [14]. Compared to mammalian P450s, bacterial enzymes have very high coupling efficiencies for native substrates [15]. Many pathways of microbial secondary metabolism contain CYPs involved in multi-step oxidation, rearrangements, epoxidations, and heteroatom oxidation [16]. For example, 18 different CYPs were found in *S. coelicolor A3(2)*. Among them, CYP158A2, CYP105N1, and CYP170A1 encode three different BGCs (i.e., T3PK synthase, non-ribosomal peptide synthase, and terpene synthase, respectively) [16,17,18]. Although the CYP450 superfamily has been studied in various *Bacillus* species associated with secondary metabolism [19], one gene encoding a unique monooxygenase (cytochrome P450BM3) that can catalyze the hydroxylation of long-chain fatty acids has been designated as CYP102A1 for *B. megaterium* [20].

This study aimed to isolate and elucidate the structure of the enriched compounds following AuNP intervention in *P. megaterium* metabolism. Bioinformatics and transcriptome analysis were then used to examine the impact of upregulation on gene expression of *csoR, CHS,* and *yjiB* that may be involved in the biosynthesis of the identified secondary metabolites.

## 2. Materials and Methods

### 2.1. Materials for RT-PCR

An RNeasy® Mini kit (cat. no. 74524) was obtained from Qiagen. BeadBug^™^ prefilled tubes with 0.1 mm silica glass beads (acid washed, 2.0 mL capacity, product no. Z763721) were from Sigma-Aldrich. A high-capacity cDNA reverse transcription kit (catalog number: 4368814), SYBR™ Green PCR master mix, and Applied Biosystems™ QuantStudio™ 3 RT-PCR system (96-well, 0.1 mL, cat. no. A28566) were obtained from Thermo Fisher Scientific.

### 2.2. Bacterial Culture of Large-Scale Fermentation with AuNP Intervention

*P. megaterium* was initially cultured for 72 h at 30 °C on an agar plate containing the following nutrients: 0.4% (*w*/*v*) glucose, 0.4% (*w*/*v*) yeast extract, 1% (*w*/*v*) malt extract, and 2% (*w*/*v*) agar in distilled water. Then, a single colony of the grown bacteria was taken from the agar plate and transferred into a 500 mL Erlenmeyer flask containing 300 mL of sterilized seed media without agar at a pH of 7.4, and the flask was incubated on an orbital shaker at 200 rpm for 72 h at 30 °C. The bacteria were further cultivated in the production media containing 10% (*v*/*v*) of the previous seed culture: 200 mL cell culture was used in a 2 L flask and incubated with or without 1.5 nM AuNPs at 30 °C in an orbital shaker incubator at 200 rpm. After six days of bacterial growth in the production media, the biomass was harvested as previously reported [21].

### 2.3. Extraction of Crude Extract

The extraction of targeted compounds after AuNP intervention in *P. megaterium* metabolism were reported previously [22]. Briefly, the biomass of bacterial growth was centrifuged, and the cell-free supernatants were obtained and then extracted with an equal volume of ethyl acetate. The organic layer was collected and then evaporated by a rotary evaporator to obtain the crude metabolite.

### 2.4. Optimization of HPLC Conditions for the Separation and Isolation of Targeted Compounds

Chromatographic separation was conducted using a reverse-phase PREP-ODS C_18_ column (250 mm × 4.6 mm, 5 µm) with a Waters® 2489 UV/Visible detector and 1525 Waters® binary HPLC pump. The initial concentration of the crude extract was 100 mg/mL. The volume of the sample in each injection was 10 µL, and analysis was carried out in triplicate. The wavelength of the maximum absorbance was determined at 240 nm. The optimal mobile phases contained isocratic elution of 5 mM ammonium acetate/HPLC water (solvent A) and MeOH/MeCN (*v*/*v*) in a 4:1 ratio (solvent B) with a total flow rate of 0.2 mL/min. Chromatographic isolation was conducted using a reverse-phase Inertsil ODS-3 Prep C_18_ column (250 mm x 10 mm, 5 µm) with a total flow rate of 0.55 mL/min and an injection volume of 50 µL. Compound 1 was collected at 59.73 -61.1 min and compound 2 at 66.7–68 min. These isolated compounds were finally dried and used later for compound identification.

### 2.5. Structural Elucidation and Characterization of Isolated Compounds by NMR, FT-IR, and MS

The ^1^H and ^13^C-NMR spectra were obtained on a Jeol Resonance 500 MHz NMR spectrometer (JNM-ECX500II, Jeol Resonance Inc., Tokyo, Japan). The isolated compounds 1 and 2 were dissolved in deuterated methanol (CD_3_OD). The IR spectrum was recorded in a Nicolet 6700 Fourier-transform infrared spectrophotometer (Thermo Fisher Scientific, Massachusetts, USA) using a KBr disc, and the spectrum was scanned from 500 to 4000 cm^−1^.

#### Spectral Analysis of Isolated Compounds

Daidzein (1): yellow crystals; MS/ESI: *m/z* 255.07*,* giving molecular formula of C_15_H_10_O_4_ [M+H]^+^. IR (KBr) max: 3431.79, 1632.78, 1596.54, 1461.76, 1239.66, 1192.05 and 840.58 cm^−1^. ^1^H NMR (500 MHz, CD3OD) δH (ppm): 8.12 (s, 1H); 8.04 (d, J = 8.8 Hz, 1H); 7.37 (d, J = 8.5 Hz, 2H); 6.92 (dd, J = 8.8, 2.2 Hz, 1H); 6.84 (dd, J = 15.8, 5.3 Hz, 3H). ^13^C NMR (125 MHz, CD3OD) δc (ppm): 178.3 (C-4), 164.7 (C-7), 159.9 (C-9), 158.8 (C-4′), 154.8 (C-2), 131.5 (C-2′), 131.5 (C-6′) 128.6 (C-5), 126.1 (C-3), 124.4 (C-1′), 118.3 (C-10), 116.6 (C-6), 116.3 (C-5′), 116.3 (C-3′), and 103.3 (C-8). NMR data were comparable to those reported in the literature and identified as daidzein [23].

Genistein (2): amorphous white powder; MS/ESI: *m/z* 271 giving molecular formula of C_15_H_10_O_5_ [M+H]^+^. ^1^H NMR (500 MHz, CD3OD) δH (ppm): 8.07 (s, 1H); 7.37 (d, J = 7.8 Hz, 2H); 6.85 (d, J = 8.6 Hz, 2H); 6.35 (d, J = 2.1 Hz, 1H); 6.23 (d, J = 2.1 Hz, 1H). ^13^C NMR (125 MHz, CD3OD) δc (ppm): 182.72 (C-4), 164.39 (C-7), 159.91 (C-5), 159.8 (C-9), 159.0 (C-4′), 154.97 (C-2), 131.54 (C-2′), 131.5 (C-6′), 124.79 (C-3), 119 (C-1′), 116 (C-3′), 116 (C-5′), 100 (C-6), 94.9 (C-8), and 80 (C-10). NMR data were comparable to those reported in the literature and identified as genistein [23].

### 2.6. RNA Extraction

Multiple gene expression was analyzed by RT-PCR for both control and AuNP-treated *P. megaterium* cultures. The bacteria were grown in an agar plate and the seed media were produced using two cultures: the control culture containing production media with 10% seeded bacteria and the AuNP-treated culture containing production media with 10% seeded bacteria and 1.5 nM AuNPs. The cells were collected by centrifugation from the medium at 24 and 48 h. The RNA was extracted from harvested and fresh cells using an RNeasy® Mini kit according to the instructions included. The concentration of the RNA was determined by a SpectraMax QuickDrop microvolume spectrophotometer (Molecular Devices®).

### 2.7. Reverse Transcriptase PCR

First-strand (single-stranded) cDNA (2 µg) was generated by the reverse transcription of RNA (0.02 µg) with a high-capacity cDNA reverse transcription kit in an ABI Veriti 384-well thermal cycler PCR instrument. RT-PCR was conducted using SYBR™ Green PCR master mix and performed in an Applied Biosystems QuantStudio 3 real-time PCR system.

All of the primers used in our experiments were purchased from Macrogen (Table 1). Primer *gyrB* was set as the endogenous control [24]. The following cycling parameters were used: 95 °C for 10 min, followed by 40 cycles of 95 °C for 15 s, and 60 °C for 60 s. The QuantStudio 3 program identified the threshold cycle (Ct) for the run based on the fluorescence values that were measured after each cycle. All samples were analyzed in quadruplicate, and the expression of target genes was calculated as relative fold values using the 2^−ΔΔCt^ method. The expression level of the target genes was normalized to that of *gyrB* (ΔCt = Ct target—Ct *gyrB*).

### 2.8. Statistical Analysis

The gene expression test was performed in quadruplicate for the RT-PCR assay and the results were averaged. Data were expressed as the mean ± standard deviation. The data were analyzed using GraphPad Prism 9.0.0. One-way ANOVA was performed for multiple comparisons in this study. Statistical significance was assumed for *p*-value < 0.05 (* *p* < 0.05).

## 3. Results and Discussion

### 3.1. Metabolic Profile of AuNP Intervention in P. Megaterium Metabolism

**Figure 1:**
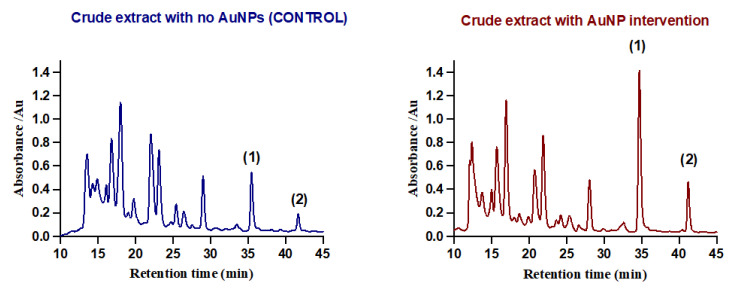
Chromatogram of optimized HPLC separation of compounds (**1**) and (**2**) from the crude extract.

The HPLC profiles of compounds 1 and 2 have previously been studied [22]. However, they were not separated well as the peaks were slightly overlapped. To avoid potential contamination of the compounds, HPLC conditions were further optimized by changing parameters such as UV detection wavelength, column, and mobile phase. As shown in Figure 1, compounds 1 and 2 were separated completely under the optimized conditions. This led to purification of compounds 1 and 2 with a large yield; the yield was 1.3 mg for compound 1 and 1.1 mg for compound 2 per 2 L of *P. megaterium* culture with AuNP intervention.

### 3.2. Isolation of Targeted Compounds

**Figure 2:**
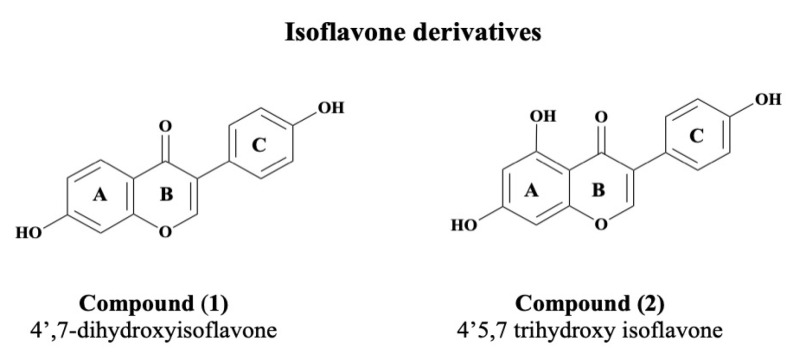
Chemical structures of compounds (**1**) and (**2**) isolated from the ethyl acetate extract of *P. megaterium* culture.

The investigation of secondary metabolites from *P. megaterium* resulted in the isolation and characterization of two compounds (Figure 2). The spectroscopic analysis of these compounds was performed using NMR, ESI-MS, and FT-IR techniques. It was found that compounds 1 and 2 were isoflavone derivatives, which are reported for the first time in this bacterium. However, numerous isoflavone derivatives were previously identified in several *Streptomyces* species [25,26,27,28]. The identity of daidzein (**1**) and genistein (**2**) was confirmed by matching with spectroscopic data in the literature [23].

### 3.3. Biosynthetic Pathway for the Two Isolated Compounds

We further explored the biosynthetic pathway for the daidzein and genistein isoflavonoids. Both daidzein and genistein are naringenin chalcones derivatives. Flavonoids and isoflavonoids are involved in phenylpropanoid biosynthetic pathways [29]. Phenylpropanoids are exclusively utilized by plants and microorganisms and are derived from the shikimate pathway, which generates essential aromatic amino acids such as phenylalanine and/or tyrosine. The biosynthetic pathway of both daidzein and genistein was already studied in plants [30]. Meanwhile, the entire biosynthesis pathway of naringenin is found in Gram-positive *S. calvuligerus* [31]. In recent years, researchers have studied the effect of NPs on *Streptomyces* metabolism to produce antibiotic secondary metabolites, with or without the induction of ROS [10,11,32]. Furthermore, CYP450 could dramatically affect microbial metabolism for the syntheis of chalcone derivatives. For instance, chalcone synthase (CHS) was found to be cotranscribed by RppA and RppB proteins in an engineered *S. griseus*. The induced transcript from the CYP450_RPP_ enzyme would be read via *rppA*–*rppB* genes and, due to this close proximity, CYP450_RPP_ was found to be involved in the synthesis and modification of flavonoids and isoflavonoids [33]. Meanwhile, quinone-forming oxygenases (MomA) belong to the cupin superfamily and are mainly responsible for the binding of one equivalent metal ion. 3,6,8-tetrahydroxynaphthalene (THN) is biosynthesized by *rppA* and its neighbor gene *CYP450-mel.* MomA catalyzes the oxygenation of THN to produce flaviolin [34]. Two decades later, the *ncs* gene encoding naringenin chalcone synthase was discovered in *S. clavuligerus*. It is required for the link to the *ncyP* gene which encodes a CYP450 oxygenase. Both are responsible for the entire synthesis of naringenin [31].

The AntiSMASH prediction has previously been discussed and suggested the naringenin molecule that appeared in the MIBiG comparison of the molecules with structural similarities correlated with those found in *S. calvuligerus* [22]. Indeed, the HPLC profile of *P. megaterium* from crude extract isolations showed daidzein (1) and genistein (2) isoflavones as shown in Section 3.2 on structure elucidation. There has never been a reported study of naringenin-related compound production and isolation in *P. megaterium*. Therefore, it would be worthwhile investigating whether the naringenin pathway and putative gene clusters in *P. megaterium* are comparable to those found in *S. clavuligerus* and whether the production of isoflavone metabolites is affected by the presence of AuNPs.

In fact, the CHS enzyme in the T3PKS cluster is well studied in plants and fungi [35]. Plant chalcone isomerase (CHI) plays a role in the production of flavonoids and catalyzes the ring fusion of chalcones, which results in the formation of the classic flavanone structure [36]. The first microbial CHI enzyme was discovered in *Eubacterium ramulus*; its role is the isomerization of naringenin chalcone to naringenin [37].

**Figure 3:**
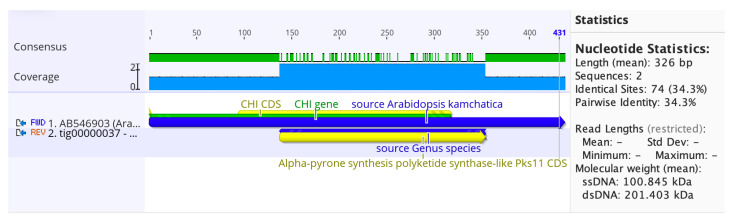
Alignment of two CDS sequences: AB546903, tig00000037—alpha-pyrone synthesis polyketide synthase-like.

We investigated the presence of the CHS enzyme in our identified bacterial isolate (*P. megaterium*). We discovered a 34.3% pairwise identity of CHS for alpha-pyrone synthesis polyketide synthase, located at contig *tig00000037* of the genomic draft compared to the *Arabidopsis lyrata subsp. Kamchatica* CHI gene, as shown in Figure 3. *P. megaterium’s* genome draft has previously been studied and identified using nanopore technology [22].

**Figure 4:**
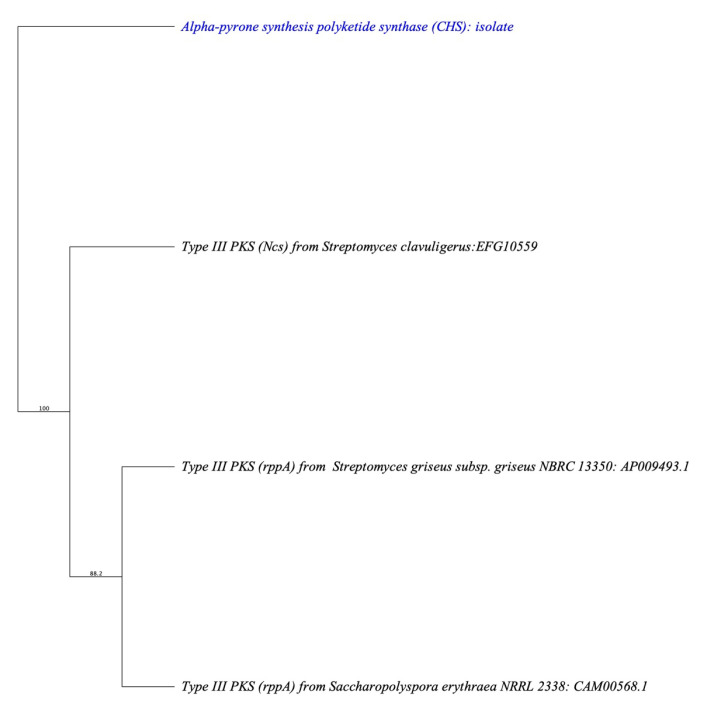
Phylogenetic relationship of four types of CHS-related genes, bootstrap values (%) presented on the branches were calculated from 1000 replications.

Then, we were more interested in finding similar genes from the chalcone synthase/stilbene synthase (CHS/STS) family in other species, such as *S. griseus, S. calvuligerus,* and *Saccharopolyspora erythraea*. We constructed a phylogenetic tree using Geneious software based on the CHS enzyme (alpha-pyrone synthesis polyketide synthase) present in the T3PKS cluster from contig *tig00000037* in comparison to the other three species mentioned earlier and chose to construct a neighbor-joining consensus tree using nucleotide alignment consensus to identify the percentage of gene identity of related genes (Figure 4).

The resulting tree (Figure 4) was a rooted tree with four taxa and seven nodes. Clades diverge at nodes, representing a speciation of gene identity from a common gene ancestor. A node represents a branching point from the common ancestral microbial CHS-related genes of all taxa described on the tree. *tig00000037* “*Alpha-pyrone synthesis polyketide synthase*” represents an outgroup to those suggested to have similar gene characteristic taxa. *tig00000037* formed a clade of the *ncs* gene from *S. clavuligerus* that is seen in the resulting phylogenetic tree. Another branch of *S. griseus* and *Saccharopolyspora erythraea* constructed a clade. All three taxa collectively form a related clade (*tig00000037*) that contains the CHS-related gene.

**Figure 5:**
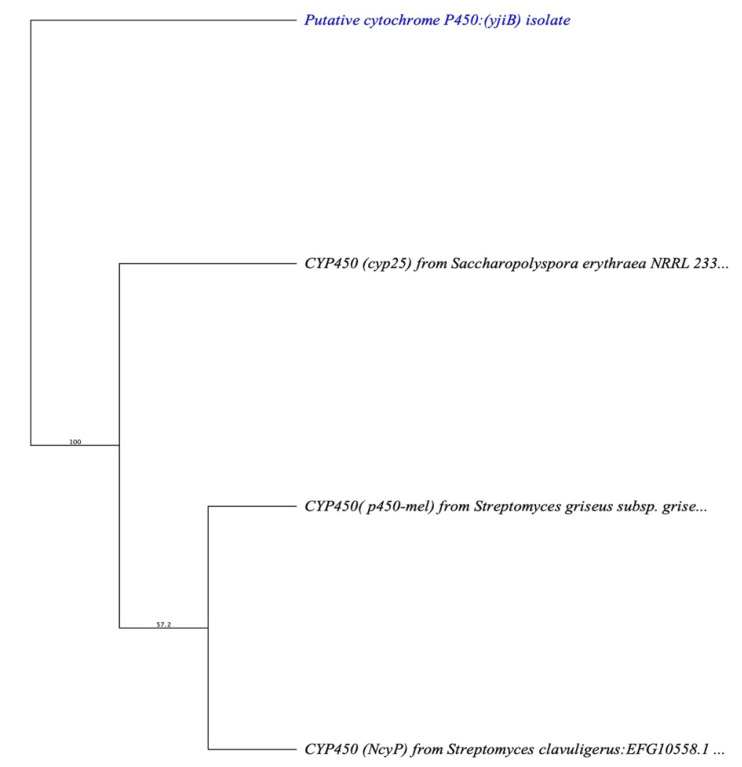
Phylogenetic relationship of four types of CYP450-related genes, bootstrap values (%) presented on the branches were calculated from 1000 replications.

Since the CYP450 oxygenase enzyme is essential in producing naringenin analogues and has always been found with the CHS enzyme, we recognized *yjiB* encoding the cytochrome p450 enzyme in the terpene cluster at contig *tig00000007* of the *P. megaterium* genomic draft. Figure 5 shows a phylogenetic tree of the *yjiB* gene in comparison to the same species mentioned above.

The resulting tree was constructed against all CYP450-related genes of the four species mentioned above to confirm whether the *yjiB* gene is very close to CYP450-related genes. The phylogenetic tree (Figure 5) showed that the *tig00000007 “yjiB”* gene represents an outgroup to the suggested CYP450-related genes and has 100% identity to a gene characteristic of a reference source for *S. griseus* to fall into one clade, despite the fact that CHS is closely related to *S. calvuligerus.*

**Figure 6:**
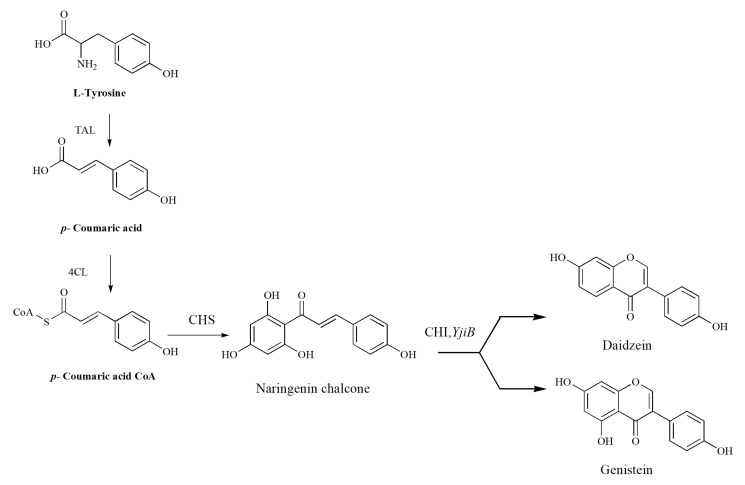
The biosynthetic pathway of daidzein and genistein and the genes anticipated in naringenin chalcone conversion.

We then performed a comparative analysis between the four species that contain both enzymes (CHS and CYP450) responsible for phenylpropanoid’s secondary metabolite biosynthesis. Our bacterial isolate shared similar gene properties, indicating that alpha-pyrone synthesis polyketide synthase and YjiB enzymes could be the main genes converting naringenin chalcone to phenylpropanoid secondary metabolite for the biosynthesis of daidzein and genistein. This suggestion indicates our strain could possess the ability to modify/catabolize flavonoids (naringenin) (Figure 6).

### 3.4. Gene Source Selection from the Genomic Draft of P. megaterium

**Figure 7:**
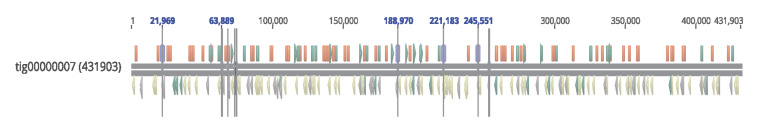
Location of genes present in *tig00000007* Terpen BGC. This annotation was extracted from Geneious version 2022.2 created by Biomatters. Available from https://www.geneious.com, accessed on 13 July 2022.

The copper-sensing transcriptional repressor (*CsoR*) gene was also present in the terpene cluster of contig *tig00000007*. The locations of the most crucial genes and the main regulatory gene that have a role in the biosynthesis of secondary metabolites were extracted from Geneious software and are shown in Figure 7. Table 2 refers to each gene and the product name.

In Table 3, we have listed the copper-related genes found in the genomic draft of *P. megaterium*, which have been extracted and identified by Geneious software. There are two copper efflux systems: one is a *CopZ-*like copper metallochaperone protein and the other is a copper-exporting P-type ATPase (*CopA*). *CsoR* and two sets of the copper chaperone are well studied in *S. lividans* [38]. This produced higher amounts of cytochrome (c) oxidases and tyrosinase in the CsoR deletion strain compared with the wild-type in response to copper stress [38]. In addition, both cytochrome (c) oxidases and quinol oxidases belong to the heme–copper family of oxygen reductases [39,40]. Thus, the presence of copper-related genes (*qoxB, qoxC, cyoC,* and cytochrome *P450*(*BM-1*)) is necessary.

### 3.5. Genes Involved in the Biosynthesis Pathway of Naringenin

According to genome mining in our previous study [22], some genes extracted from the annotation of the genomic draft could be involved in the naringenin pathway. Table S1 illustrates the location of genes putatively involved in the formation of the 4-coumaroyl-CoA precursor. Additionally, it shows the annotation of some crucial proteins involved in the biosynthesis of CHS of terpene and T3PKS clusters and defines the gene function and their percentage similarity to other known genes.

### 3.6. The Induction of csoR, CHS, and yjiB Genes by AuNPs

**Figure 8:**
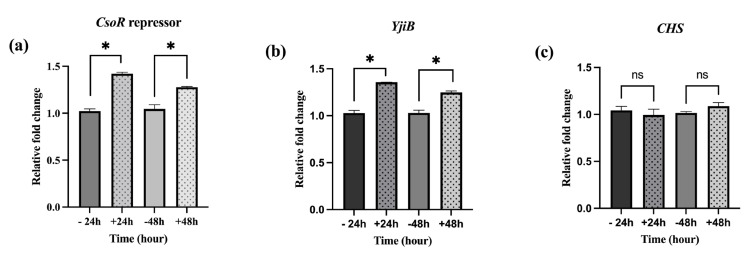
Bar graph showing the relative expression of three genes in *P. megaterium*. The fold changes in expression of *csoR* (**a**), *yijB* (**b**), and *CHS* (**c**) by RT-PCR (using *gyrB* as a reference gene). The * on the error bars indicates significant differences among the treatments (one-way analysis of variance with Tukey’s honestly significant difference; *p* < 0.05, *n* = 4; “−24h” and “−48h” are control cultures; “+24 h” and “+48 h” are AuNP intervention cultures).

A recent literature search determined that *gyrB* was the most frequently used reference gene in *P. megaterium*; this gene was used to validate RT-PCR-based gene expression experiments as a reference gene in this study. Thirty-five genes were predicted to encode naringenin biosynthesis enzymes (Table S1). Three of them (*csoR, yijB,* and *CHS*) were experimentally verified and subsequently subjected to evaluation as candidate genes. The fold expression of the control cultures (−24 h/− 48 h) and AuNP-treated cultures (+ 24 h/+ 48 h) demonstrated that the AuNP intervention significantly elevated *csoR* and *yjiB* genes in relation to the 2^−ΔΔCt^ (fold) relative gene expression as seen in Figure 8a,b. The CHS enzyme is comparable to 4-coumarate-CoA ligase, the enzyme responsible for the initial stages of naringenin synthesis. *CHS* was not affected at either 24 h or 48 h (Figure 8c).

The expression level of *csoR* and *yijB* (the gene encoding cytochrome p450 of *P. megaterium*) was upregulated, which is closely associated with the contents of daidzein and genistein. These two genes significantly responded to AuNP intervention, while CHS did not. Similar research on AgNP intervention in yeast cells showed that it has a significant impact on the genome and causes differential expression of a number of genes that are sensitive to chemical stimuli, stress, and transport processes, consequently altering the metabolism of yeast cells [9]. The genes involved in copper transport and heat shock were among those most significantly induced in response to the AgNP intervention [9].

## 4. Conclusions

In our previous research, we have studied the effect of AuNPs on genetic manipulation for enhanced secondary metabolism. This study characterized the unknown secondary metabolites isolated from *P. megaterium* after AuNP treatment and investigated the effects of AuNPs on the transcription of three genes to elucidate the mechanism of daidzein and genistein accumulation. Using a bioinformatics tool, we further investigated their biosynthetic pathway and the presence of important biosynthetic genes. Transcriptomic response analysis revealed that the AuNP intervention in *P. megaterium* metabolism induced the expression of a specific gene (*csoR*) that enhanced the expression of another gene (*yjiB*) along with the same cluster. The study’s exploration of AuNP intervention and its impact on gene expression provides insights into potential strategies for manipulating microbial metabolism. The findings could be useful in industries such as pharmaceuticals and biotechnology.

## Figures and Tables

**Table 1:** Oligonucleotide primers for metal-related, naringenin, monooxygenase, and housekeeping genes used in real-time polymerase chain reaction (RT-PCR) (the final primer concentration was 10 µM).

Target Gene	Description	Sequence (5′–3′)	Orientation	Amplicon Size (bp)	Annealing Temp (°C)
*csoR* *	Metal-sensing transcriptional repressor	TAACACCCATCGTGCGATCA GGAACAAGGAAAAGACTGCCG	Forward	80	60.8
			Reverse		61.8
*yjiB*	Putative cytochrome P450 YjiB	TGCTGCAATTTTTCGAGCGT	Forward	101	58.8
		GGCGTGCCGATAGAAGATCA	Reverse		61.6
Alpha-pyrone synthesis polyketide synthase (*CHS*)	Alpha-pyrone synthesis polyketide synthase-like Pks CDS	ACTAAATCCAGGGCCAAGAGC GTCTTCGGCAACCGTTTTGT	Forward Reverse	100	62.1 60.6
			
*gyrB*	DNA gyrase subunit B (endogenous control)	TCGTACGCTTCTTCTAACGTTCTT	Forward	91	59.1
		TTGTGAAACTTTGTAAAGAGGCGG	Reverse		59.8

* Panel of selected genes.

**Table 2:** Location of genes putatively involved in the formation of naringenin precursor.

Gene	Regulatory	Product Name	Locus_Tag	Region
-	CAP	Catabolite activator protein binding site Regulatory	-	21,969–21,990
				188,970–188,991
				221,183–221,204 245,551–245,573
*graR*	-	Response regulator protein	FJNEFGOB_01232	64,095–64,550,
*csoR*		Copper-sensing transcriptional repressor CsoR	FJNEFGOB_01240	67,832–68,095
*yjiB*	-	Putative cytochrome P450 YjiB	FJNEFGOB_01253	72,891–74,105
*gabD*	-	Succinate-semialdehyde dehydrogenase (NADP(+))	FJNEFGOB_01620	252,967–253,662
*betB*	-	NAD/NADP-dependent betaine aldehyde dehydrogenase	FJNEFGOB_01623	253,703–253,966
*gabT*	-	4-aminobutyrate aminotransferase	FJNEFGOB_01629	256,654–257,286

**Table 3:** The key genes regulating expression after copper treatment.

Copper-Related Genes	Locus_Tag	Product
*csoR 2*	FJNEFGOB_06682	Copper-sensing transcriptional repressor
*copA*	FJNEFGOB_03376 FJNEFGOB_03375 FJNEFGOB_03374 FJNEFGOB_03373 FJNEFGOB_03372	Copper-exporting P-type ATPase
*copZ*	FJNEFGOB_03369	Copper chaperone CopZ
*qoxB*	FJNEFGOB_03276	Quinol oxidase subunit 1
*qoxC*	FJNEFGOB_03278	Quinol oxidase subunit 3
*cyoC*	FJNEFGOB_03277	Cytochrome bo(3) ubiquinol oxidase subunit 3
Cytochrome P450 (BM-1)	FJNEFGOB_03253	Cytochrome P450 (BM-1)
	FJNEFGOB_03252	

## Data Availability

Not applicable.

## Supplementary Materials

The following supporting information can be downloaded at: https://www.mdpi.com/article/10.3390/cimb45080424/s1. Table S1. Blast p hits of genes putatively encoding naringenin biosynthetic enzymes, % identity, and the gene origin.
