# Supplementary material for: Priestia megaterium Metabolism: Isolation, Identification of Naringenin Analogues and Genes Elevated Associated with Nanoparticle Intervention
Source: Curr Issues Mol Biol. 2023 Aug 14;45(8):6704–16. doi: 10.3390/cimb45080424 (PMC10453022; doi:10.3390/cimb45080424)
Supplement: Supplementary file 1 [file cimb-45-00424-s001.zip › cimb-2487317-supplementary.pdf]

Supplementary Materials

Table S1. Blast p hits of genes putatively encoding naringenin biosynthetic enzymes, % identity, and the gene origin.

Enzyme	BlastP hit with	Region	Gene	% Identity	Accession	Gene origin
Malonyl-CoA-[acyl-carrier protein] trans-acylase	FJNEFGOB_07056	8,638 – 8,958	Polyketide biosynthesis protein *pksC*	64 %	CAG23952	*B. amylo-liquefaciens* gene cluster directing biosynthesis of bacillaene
	FJNEFGOB_07057			59 %		
	FJNEFGOB_07058	10,354-10,957	*pksE*	53 %		
Modular polyketide synthase	FJNEFGOB_07061	11,637 – 22,424	*pksJ*	46 %	EFG05087	*S. clavuligerus ATCC 27064*
	FJNEFGOB_07070			40 %		
	FJNEFGOB_07078			42 %		
	FJNEFGOB_07071	17,887 – 18,084	*pksN*	47 %		
	FJNEFGOB_07085	25,082 – 26,899	*pksR*	-		
	FJNEFGOB_07086	26,987 – 27,184	*pksM*	38 %		
	FJNEFGOB_07087 (Beta-ketoacyl synthase)	27,307 – 44,382	*pksL*	36 %		

	FJNEFGOB_ 07093			41 %		
	FJNEFGOB_ 07098			36 %		
	FJNEFGOB_ 07100			37 %		
	FJNEFGOB_ 07108			39 %		
	FJNEFGOB_ 07110			33 %		
	FJNEFGOB_ 07113			-		
	FJNEFGOB_ 07115			50 %		
	FJNEFGOB_ 07116			40 %		
3-oxoacyl-[acyl-carrier-protein] synthase I precursor	FJNEFGOB_ 07110			33 %		
Cytochrome P450 Penta-lenene oxygenase	FJNEFGOB_ 07801	29,639 – 30,382	*ptlI*	42 %	BAA96945	*Arabidopsis thaliana*
Betaine-aldehyde dehydrogenase	FJNEFGOB_ 07803	31,029 – 32,431	*betB*	39 %	EFG08780	*S. clavuligerus* ATCC 27064

NAD/NADP-dependent/ aldehyde dehydro-genase PuuC						
Naringenin chalcone synthase	FJNEFGOB_04952	58,588 – 58,803	-	35 %	BAB11121	*A. thaliana*
Aldehyde dehydro-genase	FJNEFGOB_04955	60,816 – 62,075	*gabD*	39%	EFG09660	*S. clavuligerus* ATCC 27064 ce
Phenylacet aldehyde dehydrogenase	FJNEFGOB_04957			37 %	EFG05910	
Phenyl-acet-aldehyde dehydro-genase	FJNEFGOB_04955					
Response regulator	FJNEFGOB_01232	64,095 – 64,550	*graR_2*	36 %	EFG08429	*S. clavuligerus* ATCC 27064
Phytoene synthase	FJNEFGOB_01234	65,125 – 65,652	-	36 %	BAB10510	*A. thaliana*
Copper-sensing transcript-	FJNEFGOB_01240	67,832 – 68,095	*csoR_1*	-	-	-

ional repressor CsoR						
Cyto-chrome P450 (P-mel) Cyto-chrome P450 hydr-oxylase	FJNEFGOB_01253	72,891 – 74,105	*yjiB_1*	31 %	EFG10558	*S. clavuligerus* ATCC 27064
ABC transporter ATP-binding protein-like/ Maltose/ malto-dextrin import ATP-binding protein	FJNEFGOB_04978	69,865 – 70,092	*malK*	41 %	BAB09414	*A. thaliana*
Ferredoxin-NADP+ reductase/ Bifunc-tional cyto-chrome P450/NAD	FJNEFGOB_07154	71,545 – 74,651	*cypD*	31 %	BAB10424	*A. thaliana*

PH–P450 reductase						
Flavin dependent oxidoreductase/ LuxAB-like protein oxygenase	FJNEFGOB_07751	-	-	100 %	EFG05499	*S. clavuligerus* ATCC 27064
Histidine kinase-like protein	FJNEFGOB_07765	-	-	43 %	BAB09274	*A. thaliana*
Acylt-ransferase	FJNEFGOB_07775	-	-	50 %	EFG06863	*S. clavuligerus* ATCC 27064
3-[(3aS,4S,7aS)-7a-methyl-1,5-dioxo-octahydro-1H-inden-4-yl] propanoyl: CoA ligase	FJNEFGOB_06920	107,950 - 106,568	*fadD3*	26.42%	COG0318	*Rhodococcus jostii*
